# Automation improves repeatability of retinal oximetry measurements

**DOI:** 10.1371/journal.pone.0260120

**Published:** 2021-12-16

**Authors:** Robert Arnar Karlsson, Olof Birna Olafsdottir, Vedis Helgadottir, Soumaya Belhadj, Thorunn Scheving Eliasdottir, Einar Stefansson, Sveinn Hakon Hardarson

**Affiliations:** 1 Faculty of Medicine, University of Iceland, Reykjavik, Iceland; 2 Faculty of Electrical and Computer Engineering, University of Iceland, Reykjavik, Iceland; 3 The National University Hospital of Iceland, Reykjavik, Iceland; 4 Faculty of Nursing, University of Iceland, Reykjavik, Iceland; 5 Eberhard Karls Universität, Tübingen, Germany; University of Florida, UNITED STATES

## Abstract

**Purpose:**

Retinal oximetry is a technique based on spectrophotometry where images are analyzed with software capable of calculating vessel oxygen saturation and vessel diameter. In this study, the effect of automation of measurements of retinal vessel oxygen saturation and vessel diameter is explored.

**Methods:**

Until now, operators have had to choose each vessel segment to be measured explicitly. A new, automatic version of the software automatically selects the vessels once the operator defines a measurement area. Five operators analyzed image pairs from the right eye of 23 healthy subjects with semiautomated retinal oximetry analysis software, Oxymap Analyzer (v2.5.1), and an automated version (v3.0). Inter- and intra-operator variability was investigated using the intraclass correlation coefficient (ICC) between oxygen saturation measurements of vessel segments in the same area of the retina.

**Results:**

For semiautomated saturation measurements, the inter-rater ICC was 0.80 for arterioles and venules. For automated saturation measurements, the inter-rater ICC was 0.97 for arterioles and 0.96 for venules. For semiautomated diameter measurements, the inter-rater ICC was 0.71 for arterioles and venules. For automated diameter measurements the inter-rater ICC was 0.97 for arterioles and 0.95 for venules. The inter-rater ICCs were different (*p* < 0.01) between the semiautomated and automated version in all instances.

**Conclusion:**

Automated measurements of retinal oximetry values are more repeatable compared to measurements where vessels are selected manually.

## Introduction

Fundus photographs are widely used to document various ocular diseases such as diabetic retinopathy [1], glaucoma [2], retinopathy of prematurity [3] and retinal vein occlusion [4]. The retinal blood vessels are visible through the optics of the eye and can be imaged with a fundus camera. Changes in retinal vessel oxygen saturation have been found in diabetic retinopathy, glaucoma, age-related macular degeneration, and other common diseases affecting the retina [5–8] as well as systemic and brain disease [9].

Measurements of retinal vessel oxygen saturation were first attempted in 1959 [10], and several groups have experimented with different approaches since then (for review, see [11–15]).

Currently, there are three commercial retinal oximeters available. One of them is the Oxymap T1 which simultaneously acquires two images of the same area of the fundus at two different wavelengths of light. The two monochromatic images are processed by specialized software, Oxymap Analyzer.

Normative oxygen saturation values in a multiethnic group of healthy subjects [16] has previously been established and the sensitivity [17] and repeatability [18] have been demonstrated. However, the Oxymap T1 is limited by the fact that measurements are semiautomated. As a result, users are required to choose each vessel segment being measured and classify it as an arteriole or venule. Manual input can lead to operator-induced variability in the measurement results. In addition, the analysis process is time-consuming and tedious.

Previously, Kumar et al. [19] reported on an automatic technique for the measurement of oxygen saturation in retinal arterioles and venules by interpreting the output of the semiautomated software for Oxymap T1. Their results showed high consistency across the dataset and indicated that an automated technique could be an accurate alternative to the semiautomated procedure.

Here we evaluate new, automatic, software for analysis of images from the Oxymap T1. The software automatically detects the vessels, classifies them as arterioles or venules, and selects the vessels once the operator defines a measurement area. This paper evaluates the effect of automation on the repeatability of measurements of retinal vessel oxygen saturation and diameter.

## Materials and methods

Normal volunteers were recrutied through an advertisement. Exclusion criteria included any retinal or optic nerve disease, eye trauma, any eye disease that affects the quality of image, diabetes mellitus, severe respiratory or cardiovascular diseases, pregnancy and breastfeeding. An ophthalmologist examined each volunteer at the eye clinic at Landspitali University Hospital.

A series of five consecutive images of the right eye were taken with the optic nerve head located in different parts of the image. Images numbered 2 and 5 had the optic nerve head located in the center and those two images were used for analysis. As other images in the series were not used in this study these images will be referred to as the *first* and *second* image respectively. All images were acquired in a dark room with the aiming light set to lowest setting. The flash intensity was set to 50 Ws. The time between flashes (images) never exceeded 30 seconds. The right eye from 23 healthy volunteers (aged between twenty and thirty years) was used for measuring.

Collection and analysis of images were done with the approval of The National Bioethics Committee of Iceland and The Icelandic Data Protection Authority All volunteers signed an informed consent.

### Retinal oximetry

The retinal oximeter (Oxymap T1; Oxymap ehf., Reykjavik, Iceland) consists of an image splitter and two digital cameras (Insight IN1800; Diagnostic Instruments Inc, Michigan, USA) attached to a fundus camera (Topcon TRC-50DX; Topcon Corporation, Tokyo, Japan). The device simultaneously acquires two images of the same area of the fundus at two different wavelengths of light, one sensitive to oxyhemoglobin (600 nm) and one isosbestic (570 nm), where oxyhemoglobin and hemoglobin absorb the same amount of light. The images are 1200 × 1600 pixels and cover a 50 field of the retina.

The light absorbance of a solution can be described in terms of optical density (OD) at wavelength λ. Optical density is defined as *OD*_λ_ = log(*I*_0λ_/*I*_λ_) where *I*_0λ_ is the intensity of the incoming light and *I*_λ_ is the intensity of the light after it has interacted with and been absorbed to some degree by the solution in question. Here *I*_0λ_ and *I*_λ_, are estimated from brightness values chosen respectively from reflected light inside vessels, and the perivascular background in the fundus images [7].

In version 2.5 *I*_λ_ is selected as the darkest pixel value along the cross-section of a vessel for each wavelength after median filtering of the image with a 3 by 3 kernel, meaning that saturation values are only calculated for points close to the center of a vessel (unless there is a central reflex from the vessel). For version 3.0, measurement points are defined for every pixel in the image within a vessel. Values of *I*_λ_ are simply the pixel value at a specific location within the image and values for *I*_0λ_ are estimated for each pixel location using inpainting [20]. In version 3.0 the effect of the central reflex on the calculated saturation values was reduced by excluding vessel pixels whose image intensity values were greater than the estimated background values at each point within the vessel.

If one of the wavelengths is sensitive to oxyhemoglobin (e.g. 600nm) and one is isosbestic (e.g. 570nm), the ratio of the optical densities ODR = *OD*_600*nm*_/*OD*_570*nm*_ at these two wavelengths will have an inverse and approximately linear relationship to the oxygen saturation (SatO2) [21]. Studies have demonstrated an artifact in the measurements caused by different diameters of the vessels [21–23] making it necessary to add a correction term.

The oxygen saturation is then calculated using *Sat*02 = *a* ⋅ *ODR* + *b* + *c* ⋅ *D* + *k* where *D* is the diameter of the vessel in pixels, and the parameters *a*, *b*, *c* and *k* can be calibrated based on measurements of arterioles and venules of healthy volunteers and assuming values from saturation measurements performed in a study with a calibrated device [23, 24]. The equation for *SatO*2 could be simplified by combining the parameters *b* and *k* but they are kept separate for consistency with the presentation in previous use [23].

Both versions detect the retinal vessels using a supervised classifier. In version 2.5 features are generated using morphological image analysis with rotated linear structuring elements which are then used to classify vessels pixels of the retinal image as belonging to a vessel or background using a linear support vector machine classifier. In version 2.5 the software determines the diameter by determining the pixels in the center of each vessel, finds a vector perpendicular to the direction of the vessel and counts the number of pixels from the center to the last pixel belonging to the vessel in each direction [25]. In version 3.0 the vessels are detected and additionally classified as arterioles or venules using serially connected U-Nets. The diameter of the vessel at each location is determined by counting the shortest path between the two edges of the vessel.

For version 2.5 of the software the calibration parameters were set to *a* = −1.28, *b* = 1.24, *c* = 0.0097 and *k* = −0.14. For version 3.0 of the software the calibration parameters were set to *a* = −1.28, *b* = 1.24, *c* = 0.0095 and *k* = −0.107.

### Analysis of oximetry images

**Fig 1:**
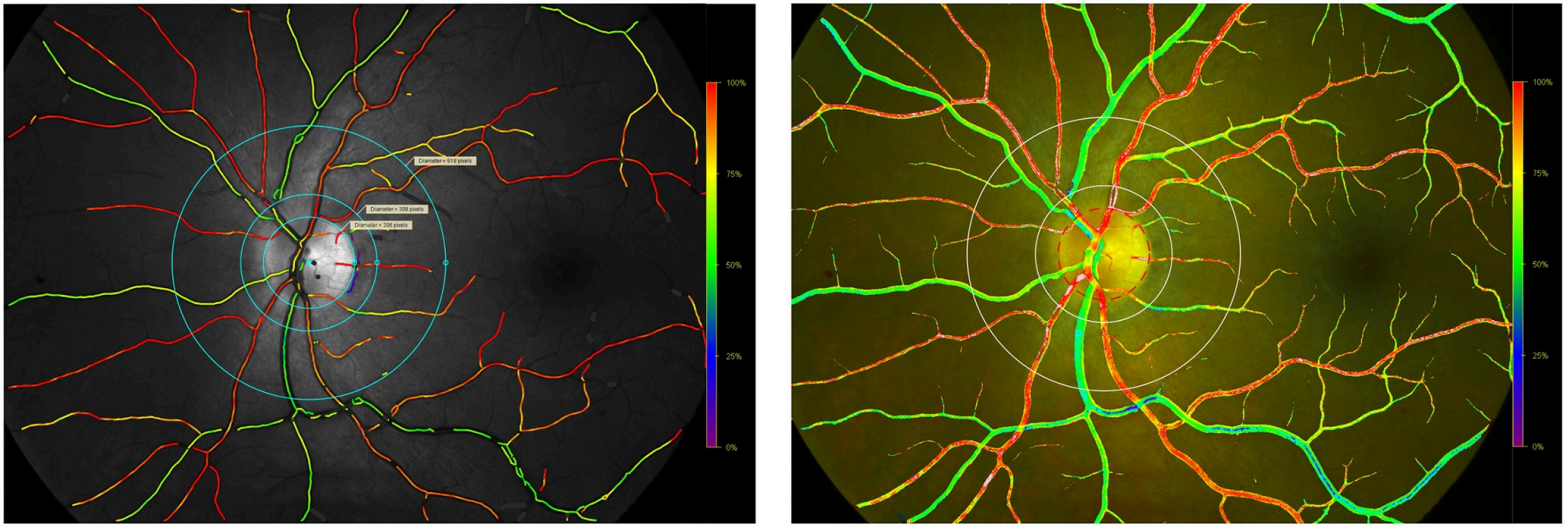
Screenshots of the same image analyzed with version 2.5 (left) and version 3.0 (right). Pseudocolor maps are generated automatically. Colors indicate the retinal vessel oxygen saturation. In healthy individuals arterioles are normally colored orange to red (approximately 90%—100% saturation). Venules are colored green to yellow-green (approximately 50%—60% saturation). Color scale is shown to the right of each image. Both software versions detect the retinal vessels automatically and select measurement points inside and outside of the vessels for automatic calculation of the retinal vessel oxygen saturation.

Images were analyzed with the latest public release of a previously validated semiautomatic software [17, 18, 25] (Oxymap Analyzer v.2.5.1, referred to as version 2.5) and new, automatic software (referred to as version 3.0). Fig 1 shows screenshots from both versions where the measurement area is defined.

The first step of the analysis process for both versions is to select the optic nerve head in the image manually. The measurement area is then defined as the area between an inner circle with a diameter 1.5 times the optic nerve diameter and an outer circle with a diameter 3.0 times the diameter of the optic nerve diameter (Fig 1). The measurement area is defined to avoid reflection of light from the retinal nerve fiber layer around the optic nerve to make the measurements more accurate. For version 2.5, segments of vessels with a diameter of 8 pixels or greater between the two circles were manually selected according to a standardized protocol (Oxymap ehf. protocol from November 2013) and classified as arterioles or venules by the operator. The mean saturation and diameter values for each segment would then be exported to an external application for further analysis. In version 3.0, the software would automatically summarize the saturation and diameter values for arterioles and venules once the operator had selected the optic nerve head in the image. Mean values of all measurement points are used, for arterioles and venules separately.

### Statistical analysis

The software versions are compared in several ways.

The mean and standard deviation of the saturation and diameter values are given for arterioles and venules for both versions calculated using the measurement values from the first image for all operators. The as there are repeated measurements for each image the standard deviation is estimated using variance components from the analysis of variance (ANOVA) [26] for each version separately where the subject and operator are considered random effects. The mean values for each version and parameter is evaluated using a mixed effects ANOVA model with subject and operators as random effects and version as a fixed effect with p-values for F-tests based on Satterthwaite’s method [27].

Intraclass correlation coefficients (ICCs), and their 95% confidence intervals, are used to assess the intra (*ICC*_*intra*_.) and inter-operator (*ICC*_*inter*_) reliability using a two-way random factorial absolute agreement ANOVA model [28]. When interpreting reliability using the ICC scores, “values less than 0.5 are indicative of poor reliability, values between 0.5 and 0.75 indicate moderate reliability, values between 0.75 and 0.9 indicate good reliability, and values greater than 0.90 indicate excellent reliability” [28]. We, therefore, analyze the repeatability of measurements with the two versions using the 95% limits of agreement method (LoA) [29]. The repeated measurements are for five operators, separately for oxygen saturation and diameter, and are used to estimate the LoA for the two versions. As there are observations from several operators for each image the LoAs are calculated for multiple replicates with 95% CIs calculated using the MOVER method [30].

Finally, the time in seconds taken to analyze an image is measured for both versions. The mean and standard deviation are calculated. Statistical significance of the difference is evaluated using a two-tailed, paired t-test.

Statistical analysis and generation of plots and graphs was performed in R version 3.6.1 [31] using irrICC [32] version 1.0, ggplot2 [33] version 3.3.2 and boot [34, 35] version 1.3–25.

## Results

**Fig 2:**
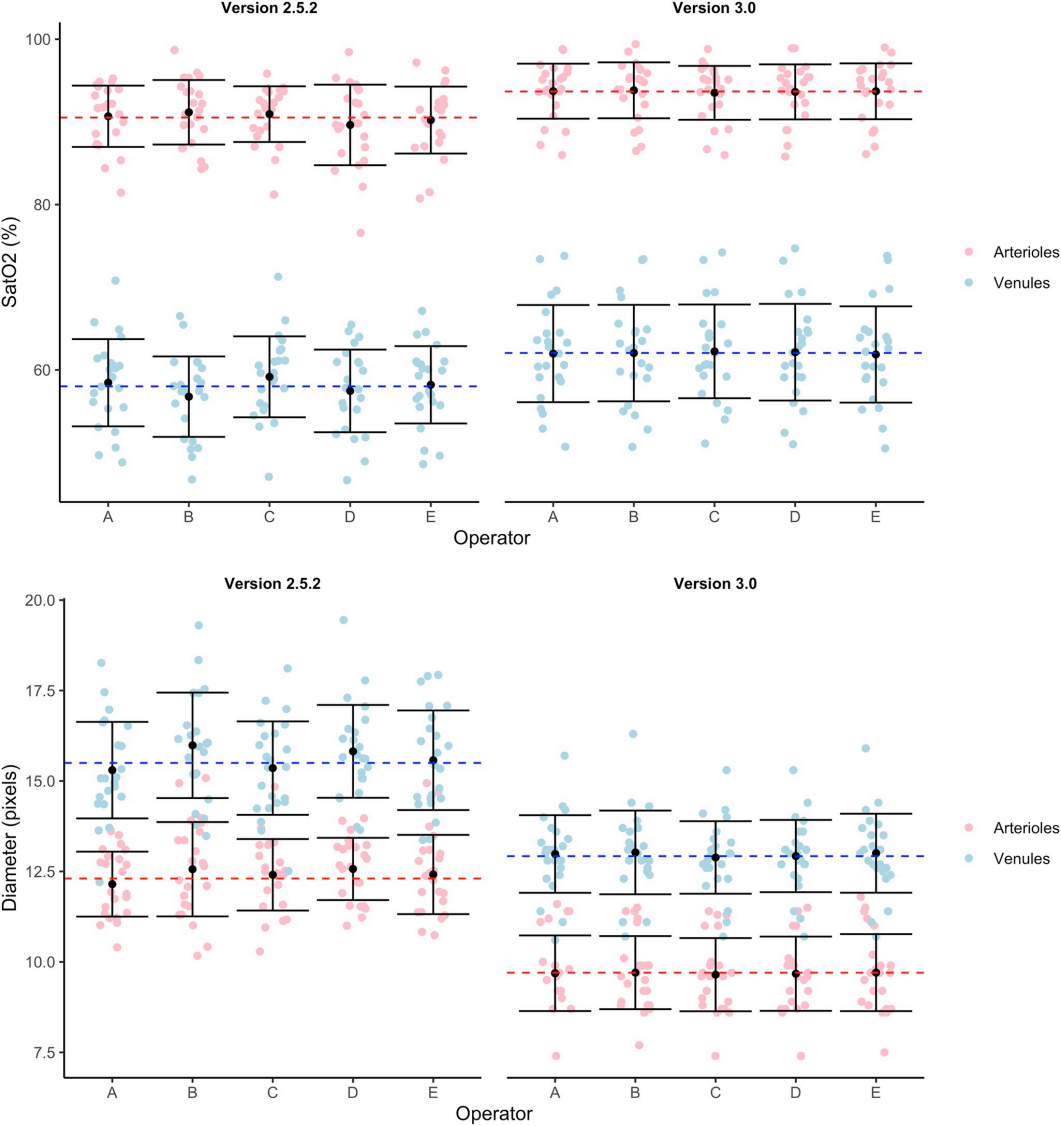
Measured vessel saturation (top) and diameter (bottom) values from the first image for the two versions and all operators. Light red and blue points show the mean value from one image, dashed red and blue lines show the global mean values for arterioles and venules, respectively. Black dots show the mean, and error bars show the standard deviation around the mean for each operator and vessel type.

Fig 2 shows saturation and diameter measurements for the first image of each participant. Oxygen saturation in retinal arterioles was different between analysis software; 90.7%±3.9% for the semiautomated version 2.5 and 93.7%±3.4% for the automatic version 3.0 (*p* < 0.0001, mean±SD). Venular oxygen saturation was lower measured with version 2.5, 58.9%±5.2% compared to version 3.0; 62.3%±5.8%, (*p* < 0.0001, mean±SD).

For diameter measurements, arteriolar diameter was decreased for version 3.0 compared with version 2.5 (9.7±1.1 pixels vs. 12.3±1.1 pixels; *p* < 0.0001) as well as the venular diameter (12.9±1.4 pixels vs. 15.5±1.0 pixels; *p* < 0.0001).

**Fig 3:**
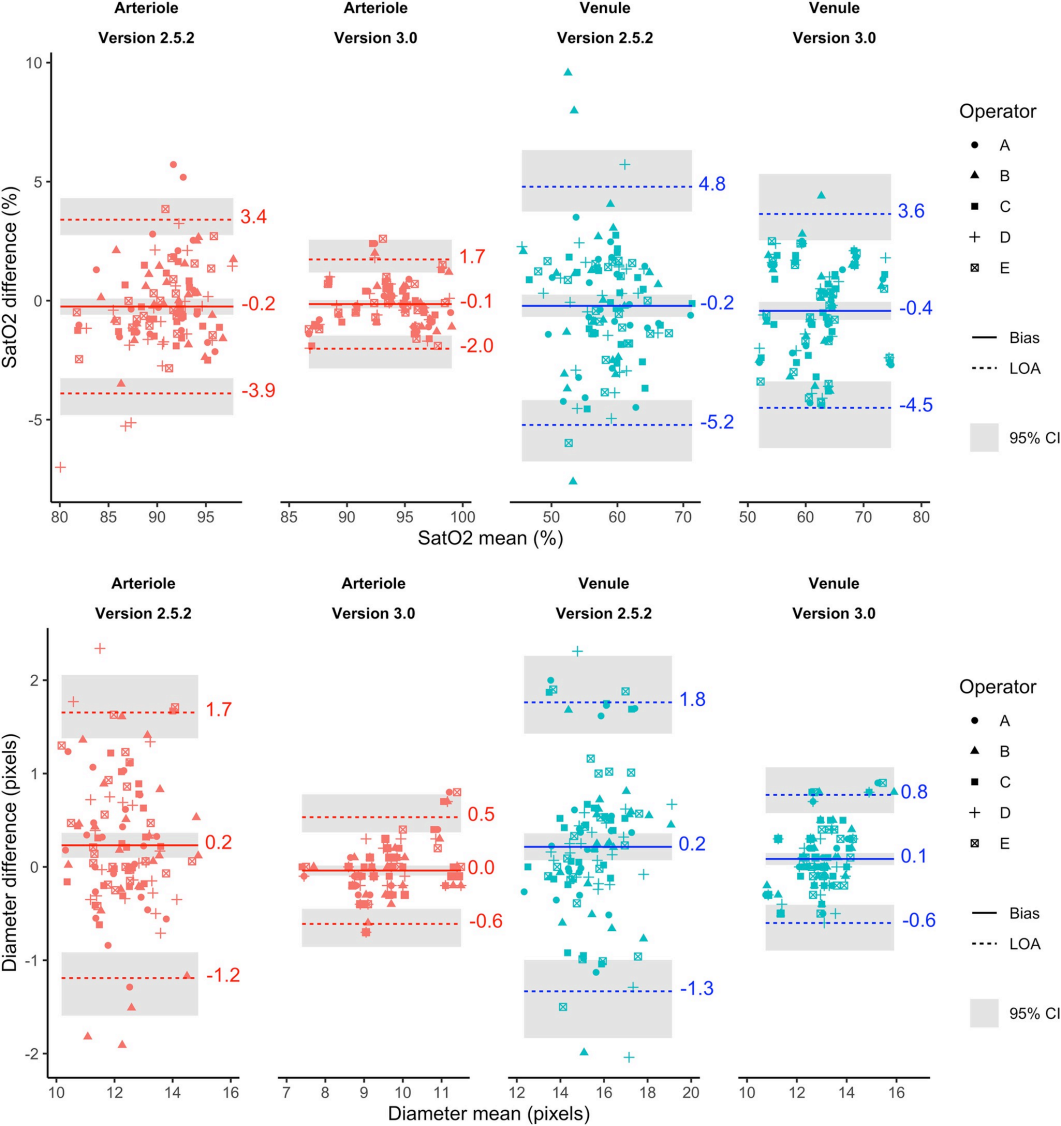
Analysis of repeatability of retinal vessel oximetry (top) and diameter (bottom). The same area in two images acquired within a short time interval is measured using the two software versions for arterioles and venules. The vertical axis shows the difference between two saturation and diameter measurements in the same area (first image minus second image), and the horizontal axis shows their mean. The solid line in the middle shows the mean difference, and the other two broken lines show Limits of Agreement (LoA) or the two standard deviations of the difference. Results are colored red for arterioles and blue for venules. Grey shaded areas show the 95% confidence interval around the bias and LoA.

Fig 3 shows Bland-Altman plots for saturation and diameter measurements.

Table 1 shows intra and inter-rater reliability when measuring vessel oxygen saturation.

**Table 1:** Intra and inter-rater reliability when measuring vessel oxygen saturation.

	Version	ICC_intra_ [95% CI]	ICC_inter_ [95% CI]
Arterioles	2.5	0.80 [0.70, 0.90]	0.81 [0.71, 0.90]
Arterioles	3.0	0.97 [0.95 0.99]	0.97 [0.95, 0.99]
Venules	2.5	0.80 [0.68 0.90]	0.84 [0.75, 0.92]
Venules	3.0	0.96 [0.94 0.98]	0.96 [0.94, 0.98]

Intra and inter-rater reliability (ICC) when measuring vessel oxygen saturation using the two versions. Measurements from the same area of 23 right eyes in 23 subjects.

For arterioles measured with version 2.5 the intra and inter-rater reliability is moderate to good [28]. For venules measured with version 2.5 the intra-rater reliability is moderate to good and the inter-rater reliability is good to excellent. For arterioles and venules measured with version 3.0 the intra and inter-rater reliability is excellent.

Table 2 shows the intra and inter-rater reliability when measuring vessel diameter.

For arterioles and venules measured with version 2.5 the intra and inter-rater reliability is moderate to good. For arterioles and venules measured with version 3.0 the intra and inter-rater reliability is excellent.

**Table 2:** Intra and inter-rater reliability when measuring vessel diameter.

	Version	ICC_intra_ [95% CI]	ICC_inter_ [95% CI]
Arterioles	2.5	0.71 [0.58, 0.84]	0.73 [0.60, 0.85]
Arterioles	3.0	0.97 [0.96, 0.99]	0.97 [0.96, 0.99]
Venules	2.5	0.71 [0.56, 0.84]	0.76 [0.65, 0.87]
Venules	3.0	0.95 [0.91, 0.98]	0.95 [0.92, 0.98]

Intra and inter-rater reliability (ICC) when measuring vessel diameter using the two versions. Measurements from the same area of 23 right eyes in 23 subjects.

The analysis time per image was 617±187 seconds for version 2.5 and 29±7 seconds for version 3. Using version 3.0 the analysis of images was significantly faster than for version 2.5 (p = 0.032).

## Discussion and conclusions

This study compares two versions of software capable of measuring retinal vessel oxygen saturation and vessel diameters. Vessels from the same area were measured in two images taken of the same subject with a short time interval. One version (v3.0) automatically selects the vessels to be measured and classifies them as arterioles or venules, while the other version (v2.5) requires operator input for these tasks.

Version 3.0 measures higher oxygen saturation values for both arterioles and venules than version 2.5 but the variability between measured subjects is similar or slightly lower for version 3.0.

The limits of agreement analysis for oxygen saturation indicate that repeatability is substantially improved for arterioles but less so for venules. The intra and inter-rater ICCs indicate that repeatability, and agreement between operators is improved for version 3.0 compared with version 2.5.

When measuring vessel diameter version, 3.0 performs substantially better for both arterioles and venules compared with version 2.5. The lower measured diameter in version 3.0 versus version 2.5 can be attributed to superior vessel detection where the boundaries of vessels are more precisely located. Measurements with version 3.0 therefore include some of the smaller vessels that were not detected by version 2.5. As this might lead one to suspect that the lower LoA was simply an artifact of these lower measurement values, the diameter measurements were also compared after the values from version 3.0 had been scaled to have the same mean value as the diameter measurements from version 2.5. Even after this adjustment, the widest LoA for version 3.0, taking into account the outer limits of the 95%CI, was narrower than the narrowest LoA for version 2.5, where the inner limits of the 95% CI were used. We, therefore, conclude that there is substantial evidence that the diameter measurements for version 3.0 are more repeatable than for version 2.5.

This study has several limitations which should be addressed in follow up studies. Here, only images from young, healthy volunteers were used. While anecdotal evidence shows that the automated software works well with images from patients suffering from conditions such as CRVO and diabetic retinopathy the performance should be validated beyond the healthy eye.

Additionally, the two software versions process the images in different ways which can explain the differences in the measured values for both oxygen saturation and vessel diameter. Based on images processed by both software versions the authors believe that the vessel detection in version 3.0 performs as well as, and often substantially better, than the vessel detection for version 2.5. It is therefore the opinion of the authors that version 3.0 gives vessel diameter estimates which are closer to the true values. The method for averaging vessel diameters is also less convoluted for version 3.0. This will however need to be verified in future studies where the performance of the two methods is compared against reference diameter values acquired by human experts.

For the oxygen saturation the two software versions give different values for both arteries and veins. This difference can be attributed to several factors. First, because of the apparently superior vessel detection there are some vessels included in the measurement for version 3.0 which are not included in the measurements for version 2.5. These are however smaller vessels whose measurement values are typically more variable. This would therefore be unlikely to give a more favorable result for variability with version 3.0. Secondly because of the differences between the two software versions in measured diameters values, number of vessels detected and points used for saturation calculation different calibration parameters were derived for version 3.0. While both software versions measure diameter and oxygen saturation values using the same general approaches the image processing is different between the software versions. Pixels within a vessel were for instance binned within vessels for version 2.5 but not for version 3.0 and perivascular background was estimated in a with a different method in the two software versions. Finally, the same points are not used for the calculation between the two versions. However, as obtaining ground truth measurements of the oxygen saturation in the retinal vessels of humans is difficult and therefore these measurement values are provided as relative. While the current study shows that measurements of oxygen saturation values and diameter values are repeatable further comparative studies are needed, for instance where healthy individuals breathe 100% oxygen [36] to compare the sensitivity of the two software versions to changes in retinal oxygen saturation. Further studies with a larger set of healthy volunteers as well as those suffering from retinal diseases are needed to test if version 3.0 gives as good or a better idea of the retinal oxygen saturation compared to version 2.5 and to ensure that the method proposed by version 3.0 is robust against artifacts which are potentially introduced with the new methodology.

In conclusion, while both versions of the software evaluate the oxygen saturation in a similar manner, the measured values are slightly different, which means that results from the two software versions are not directly comparable. The automatic software (Version 3.0) gives more consistent results, both for repeated measurements and between operators, than the semiautomated software. Finally, the automated measurements are substantially faster than the semiautomated measurements.

## Supporting information

S1 File Specific data for all individuals and operators. (ZIP)
